# Concise and Gram-Scale Total Synthesis of Lansiumamides A and B and Alatamide

**DOI:** 10.3390/molecules24203764

**Published:** 2019-10-19

**Authors:** Ran Lin, Xi Lin, Qian Su, Binbin Guo, Yanqin Huang, Ming-An Ouyang, Liyan Song, Huiyou Xu

**Affiliations:** Key Laboratory of Biopesticide and Chemical Biology, Ministry of Education, College of Plant Protection, Fujian Agriculture and Forestry University, Fuzhou 350002, China; lrsummer@163.com (R.L.); lynsey_1@163.com (X.L.); fafusuqian@163.com (Q.S.); bgleet@sina.com (B.G.); hyqviolet@163.com (Y.H.); minganouyang@163.com (M.-A.O.)

**Keywords:** total synthesis, lansiumamide, alatamide, copper-catalyzed buchwald coupling

## Abstract

The total synthesis of potent anti-obesity lansiumamide B was accomplished in four steps using commercially available materials. The synthetic strategy, featured with copper-catalyzed Buchwald coupling, is concise, convergent, practical and can be carried out on a one-gram scale. This approach could give either *Z*- or *E*-configured enamide moiety in natural products with absolute stereocontrol and was applied in the total synthesis of natural products.

## 1. Introduction

**Figure 1:**
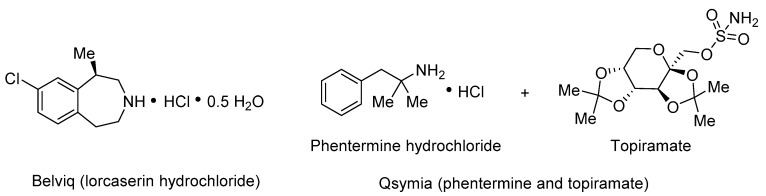
Molecular structure of Belviq and Qsymia.

Obesity is characterized by abnormal or excessive fat accumulation in adipose tissue with deleterious effects on human health. The prevalence of severe obesity has increased in recent decades [1]. The association of obesity with type 2 diabetes has been recognized for decades, and the major basis for this link is the ability of obesity to engender insulin resistance [2]. Both obesity and diabetes generate immense health care costs, and cause severe morbidity and mortality worldwide [3]. Thus, anti-obesity agents (Figure 1) would provide a first line of defense and become a powerful weapon against obesity as well as diabetes.

In the search for new anti-obesity natural products, Lin [4] and co-workers reported in 2017 on lansiumamide B (**2**) isolated from the seeds of *Clausena lansium*, which has demonstrated a variety of pharmacological activities and has been widely used in Chinese folk medicine for the treatment of acute and chronic gastro-intestinal inflammation and ulcers [5] (Figure 1). In contrast to the FDA approved anti-obesity drugs possessing unpleasant adverse effects [6], the natural source of lansiumamide B (**2**), the edible fruit of *Clausena lansium*, called wampee, is highly aromatic and pleasantly sweet. Noticeably, lansiumamide B (**2**) was found to exhibit potent activity against fat mass gain through suppressing lipogenesis. Moreover, lansiumamide B (**2**) significantly enhanced insulin sensitivity and thus effectively improved high fat diet-induced insulin resistance [4]. Therefore, lansiumamide B (**2**) with a skeleton distinct from those of all known anti-obesity agents (cf., Belviq and Qsymia, Figure 1) holds great potential as a promising anti-obesity drug/lead.

**Figure 2:**
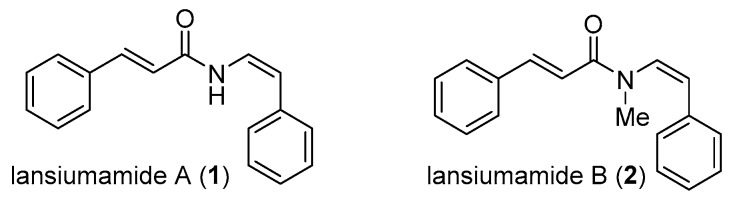
Molecular structure of lansiumamides A and B.

Lansiumamides A (**1**) and B (**2**) were firstly isolated in 1989 [7] (Figure 2). In the last decade, lansiumamide B (**2**) was also found to display anti-inflammatory [8] and anti-necrosis activities [9]. Structurally, lansiumamide A and B were cinnamamide derivatives containing *Z*-enamide. The *Z* configuration of enamide, which is critical to the potent bioactivities, might pose some synthetic challenges.

**Scheme 1:**
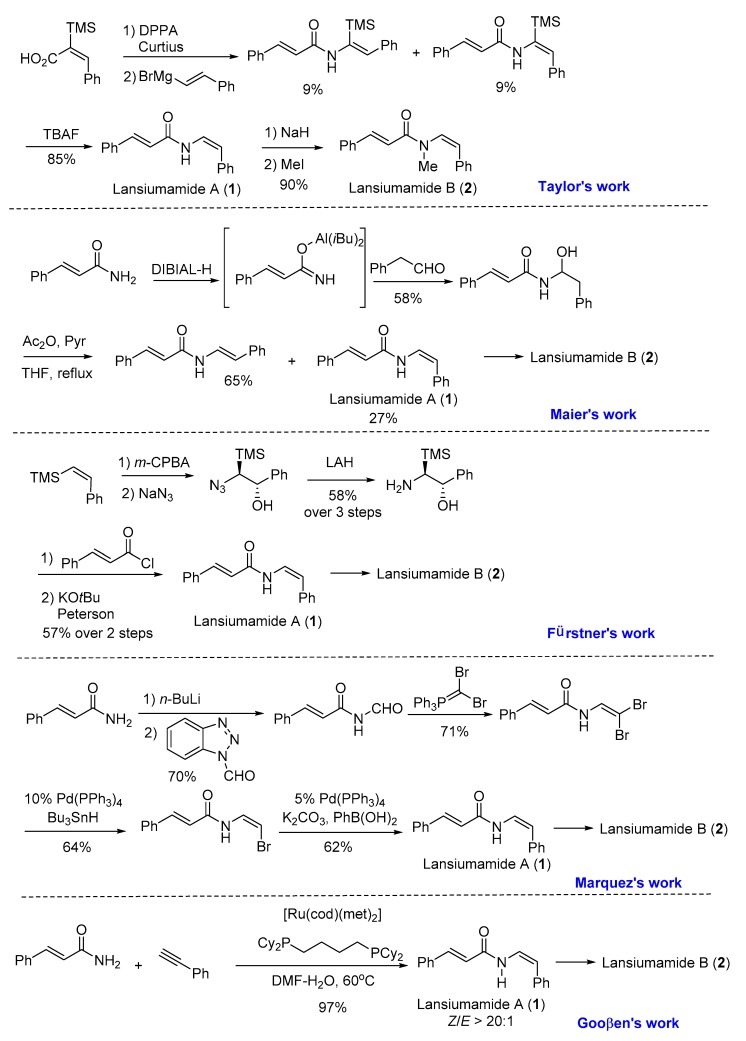
Previous work for total synthesis of lansiumamides A and B.

Lansiumamides A and B have received considerable attention from the synthetic community (Scheme 1). Taylor [10] and Maier [11] conducted the pioneering work of a total synthesis of lansiumamides A and B. In these syntheses, the enamide moiety was obtained as a mixture of *Z*/*E* isomers. Fürstner [12] completed the first stereoselective synthesis of lansiumamide A by a Peterson reaction manifold in 2001. Marquez [13] achieved the total synthesis of lansiumamide A and B by taking advantage of the dihalo-olefination of *N*-formaylimides and the subsequent stereoselective reduction to afford *Z*-configured vinyl bromide. It is noteworthy that Gooβen [14] reported the single-step stereoselective synthesis of lansiumamides A and B via ruthenium-catalyzed hydroamidation of phenylacetylene and cinnamide.

We have used Gooβen’s protocol to synthesize lansiumamide B and generate lansiumamide B derivatives as antifungal agents [15]. Although the overall yield of the two-step synthesis of lansiumamide B is satisfactory (78%), the yield for the two-step synthesis decreased rapidly on scale-up above 1 mmol. Hence, a more practical and scalable approach with an easily accessible catalyst and reagent is still highly desirable to construct the enamide moiety in a stereospecific fashion. The combination of the potent anti-obesity activity coupled with the structural novelty prompted us to undertake synthetic studies of lansiumamides A and B. Herein, we report a concise, convergent, and gram-scale total synthesis of lansiumamides A and B, which was enabled by copper-catalyzed Buchwald coupling [16].

**Scheme 2:**
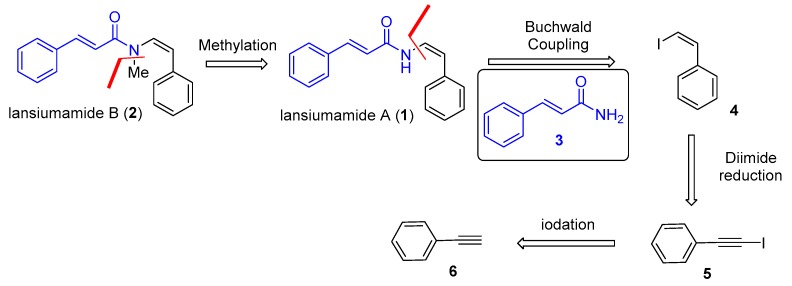
Synthetic plan for lansiumamides A and B.

Inspired by the fact that the configuration of enamide could be completely controlled by the configuration of vinyl iodide in the copper-catalyzed Buchwald coupling [16], we proposed a stereospecific synthetic strategy for lansiumamides A and B (**1** and **2**) as depicted in Scheme 2. Lansiumamide B (**2**) could be easily obtained from Lansiumamide A (**1**) via methylation. We envisioned that the copper-catalyzed Buchwald coupling [16] of cinnamamide (**3**) would provide the *Z*-configured enamide found in lansiumamides A and B, when the (*Z*)-(2-iodovinyl)benzene (**4**) bearing *Z*-configuration was employed as the corresponding vinyl halide. The stereochemistry of the newly formed enamide in lansiumamide A arises from the configuration of the vinyl iodide used. (*Z*)-(2-iodovinyl)benzene (**4**) could be synthesized readily and stereospecifically via diimide reduction [17]. The easy preparation of (**4**) from the commercially available (**6**) via several known transformations encourages us to explore this synthetic sequence.

## 2. Results and Discussion

**Scheme 3:**
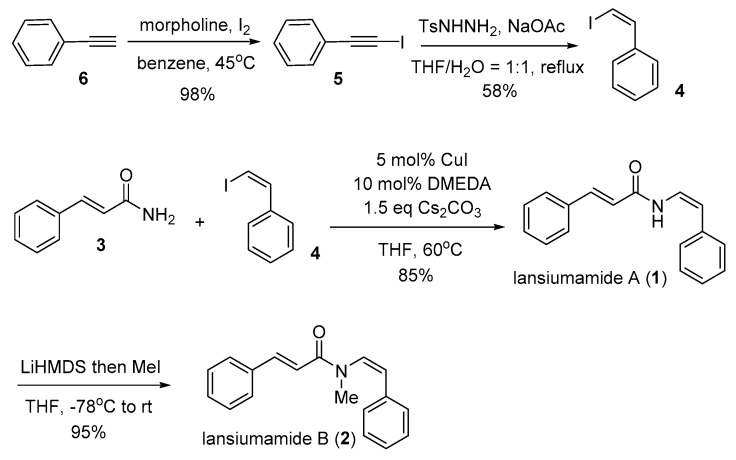
Total synthesis of lansiumamides A and B.

Our synthesis (Scheme 3) began with the preparation of (iodoethynyl)benzene (**5**) from commercially available phenylacetylene (**6**) by using the protocol developed by Lubin–Germain and Augé [18], followed by a diimide reduction [17] to afford (*Z*)-(2-iodovinyl)benzene (**4**). With a reliable supply of (*Z*)-(2-iodovinyl)benzene (**4**) in hand, we reached the key stereospecific copper-catalyzed Buchwald coupling [16]. To our delight, the reaction had run very well with absolute stereocontrol and afforded the expected lansiumamide A (**1**) in high yield as a single double bond isomer. The total synthesis of lansiumamide B (**2**) was accomplished via lithium bis(trimethylsilyl)amide (LiHMDS)-mediated methylation. It is noteworthy that the synthetic route is reliable and gram scale, for the desired lansiumamide B (**2**) was obtained in gram scale. All spectroscopic data for our synthetic samples were in good agreement with those reported for the corresponding natural products.

**Figure 3:**
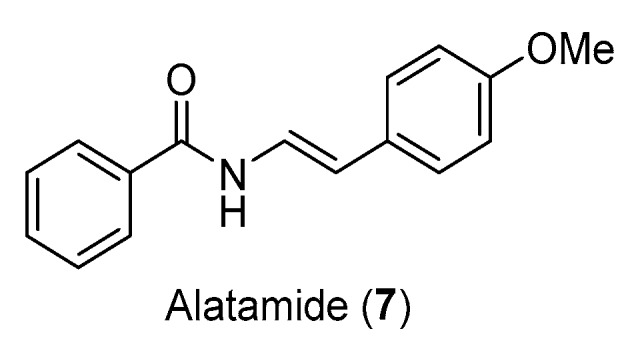
Molecular structure of alatamide.

Next, our attention turned to the application of our protocol in the total synthesis of natural products containing (*E*)-enamide. We selected alatamide (**7**), which was isolated from the aerial parts of the plant *Piper guayranum* [19,20] and contains a 4-methoxyphenyl substituted (*E*)-enamide unit (Figure 3), as the example to present our synthetic sequence.

**Scheme 4:**
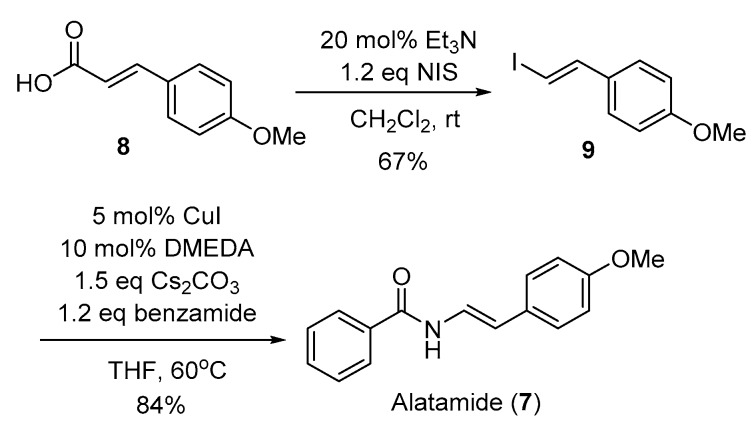
Total synthesis of alatamide.

Our synthesis of alatamide (**7**) (Scheme 4) commenced with a modified Hunsdiecker–Borodin reaction [21] of commercially available 4-methoxy-cinnamic acid (**8**) to provide (*E*)-1-(2-iodovinyl)-4-methoxybenzene (**9**) for the key coupling reaction. Gratifyingly, under the identical coupling conditions for the syntheses of lansiumamides A and B, benzamide underwent copper-catalyzed coupling [16] with (*E*)-1-(2-iodovinyl)-4-methoxybenzene (**9**) to furnish the alatamide (**7**) in good yield as a single double-bond isomer. All the spectroscopic data of our synthetic sample were identical to the reported data of alatamide. In addition, the final product was also secured in gram scale.

## 3. Materials and Methods

### 3.1. General

Reactions were carried out in oven or flame-dried glassware under a nitrogen atmosphere, unless otherwise noted. Tetrahydrofuran (THF) was freshly distilled before use from sodium using benzophenone as an indicator. Dichloromethane was freshly distilled before use from calcium hydride (CaH_2_). All other anhydrous solvents were dried over 3 Å or 4 Å molecular sieves. Solvents used in workup, extraction, and column chromatography were used as received from commercial suppliers without prior purification. Reactions were magnetically stirred and monitored by thin layer chromatography (TLC, 0.25 mm) on Liangchen pre-coated silica gel plates. Flash chromatography was performed with silica gel 60 (particle size 0.040–0.062 mm) supplied by Liangchen. Infrared spectra were collected on a Bruker model TENSOR27 spectrophotometer. ^1^H and ^13^C NMR spectra were recorded on a Bruker AVIII-400 spectrometer (400 MHz for ^1^H, 100 MHz for ^13^C). Chemical shifts are reported in parts per million (ppm) as values relative to the internal chloroform (7.26 ppm for ^1^H and 77.16 ppm for ^13^C). Abbreviations for signal coupling are as follows: s, singlet; d, doublet; t, triplet; q, quartet; m, multiplet. Benzene was used as solvent in Section 3.2.1 (CAUTION! STRONG CARCINOGENIC ACTIVITY). The visualization of NMR spectra could be found in the Supplementary Materials.

### 3.2. Total Synthesis of Lansiumamides A and B

#### 3.2.1. Synthesis of (Iodoethynyl)Benzene (**5**)

Iodine (2.80 g, 11.0 mmol) and morpholine (2.61 g, 2.62 mL, and 30.0 mmol) were dissolved in benzene (12 mL) and the solution was stirred at RT for 30 min. Phenylacetylene (**6**) (2.25 g, 10.0 mmol) in benzene (18 mL) (CAUTION! STRONG CARCINOGENIC ACTIVITY) was added dropwise and the mixture was stirred at 45 °C for 24 h. The suspension was filtered and the residue was washed with Et_2_O (2 × 20 mL). The combined organic layers were washed with saturated aqueous solutions of NH_4_Cl (20 mL), NaHCO_3_ (20 mL), and H_2_O (20 mL). The organic layer was dried over MgSO_4_ and filtered. The solvent was removed under reduced pressure and the resulting residue was purified by flash chromatography on silica gel using eluents (petroleum ether) to afford the desired (iodoethynyl)benzene (**5**) [22] (2.23 g, 98% yield) as a yellowish oil. ^1^H NMR (400 MHz, CDCl_3_) δ: *δ* = 7.47–7.43 (m, 2H), 7.36–7.30 (m, 3H). ^13^C NMR (100 MHz, CDCl_3_) δ: 132.4 (2×C), 128.9, 128.4 (2×C), 123.5, 94.2, 6.4.

#### 3.2.2. Synthesis of (*Z*)-(2-Iodovinyl)Benzene (**4**)

To a solution of (iodoethynyl)benzene (**5**) (2.23 g, 9.8 mmol) in THF (20 mL) water (20 mL) was added 4-Methylbenzenesulfonhydrazide (3.65 g, 19.6 mmol) and sodium acetate (2.41 g, 29.4 mmol). The reaction mixture was stirred and refluxed vigorously. After TLC analysis indicated complete consumption of the starting material (12 h), the reaction mixture was cooled to room temperature and then extracted with Et_2_O (3 × 20 mL). The combined organic fractions were washed with brine and dried with anhydrous Na_2_SO_4_. The solvents were evaporated under reduced pressure and the resulting residue was purified by flash chromatography on silica gel using eluents (petroleum ether) to give the desired (*Z*)-(2-iodovinyl)benzene (4) [23,24] (1.31 g, 58% yield) as a colorless oil. ^1^H NMR (400 MHz, CDCl_3_) δ: *δ* = 7.66–7.63 (m, 2 H), 7.43–7.36 (m, 3H), 7.33 (d, *J* = 8.8 Hz, 1H), 6.58 (d, *J* = 8.8 Hz, 1H). ^13^C NMR (100 MHz, CDCl_3_) δ: 138.7 (2×C), 136.8, 128.6 (2×C), 128.5, 128.3, 79.5.

#### 3.2.3. Synthesis of Lansiumamide A (**1**)

A Schlenk tube was charged with CuI (48 mg, 0.25 mmol, 5 mol%), Cs_2_CO_3_ (2.44 g, 7.5 mmol), and cinnamamide (**3**) (0.882 g, 6.0 mmol), and evacuated and backfilled with argon. *N*,*N*′-Dimethylethylenediamine (54 μL, 0.50 mmol, 10 mol%), (*Z*)-(2-iodovinyl)benzene (**4**) (1.15 g, 5.0 mmol) and THF (10.0 mL) were added under argon. The Schlenk tube was sealed, immersed in a preheated oil bath; the reaction mixture was stirred at the 60 °C until the complete consumption of starting material was observed by TLC (3 h). The reaction vessel was removed from the oil bath and the resulting suspension was allowed to reach room temperature, then, it was filtered through a plug silica gel eluting with ethyl acetate (50 mL). The filtrate was concentrated and the residue was purified by flash chromatography on silica gel using eluents (petroleum ether/ethyl acetate = 5/1) to furnish lansiumamide A (**1**) as yellowish solid (1.06 g, 85% yield). Mp = 120–122 °C. (lit. 121–123 °C) [7] ^1^H NMR (400 MHz, CDCl_3_) δ: 7.85 (br, 1H), 7.73 (d, *J* = 15.6 Hz, 1H), 7.53–7.49 (m, 2H), 7.44–7.26 (m, 8H), 7.13 (dd, *J* = 11.6, 10.0 Hz, 1H), 6.40 (d, *J* = 15.6 Hz, 1H), 5.84 (d, *J* = 10.0 Hz, 1H). ^13^C NMR (100 MHz, CDCl_3_) δ: 163.3, 143.2, 135.9, 134.6, 130.3, 129.3 (2×C), 129.0 (2×C), 128.2 (2×C), 128.1 (2×C), 127.2, 122.4, 119.6, 110.8. Anal. calcd for C_17_H_15_NO: C, 81.90; H, 6.06; N, 5.62. Found: C, 81.80; H, 6.18; N, 5.51.

#### 3.2.4. Synthesis of Lansiumamide B (**2**)

A flame-dried round bottle under argon atmosphere was charged with lansiumamide A (**1**) (1.03 g, 4.13 mmol) and anhydrous tetrahydrofuran (THF, 15 mL). The solution was cooled to −78 °C and lithium hexamethyldisilazide (LiHMDS, 5 mL, 1.0 M in THF, 5.0 mmol) was added dropwise. The reaction mixture was warmed to 0 °C gradually, stirred for an additional 30 min and then cooled to −78 °C. Iodomethane (0.63 mL, 10 mmol) was added dropwise to the cold solution. The reaction mixture was then allowed to gradually warm to room temperature and stirred overnight. Aqueous saturated NH_4_Cl solution (5 mL) was added under vigorous stirring to quench the reaction. The volatiles (mainly THF) were removed under reduced pressure and the aqueous phase was extracted with diethyl ether (3 × 10 mL). The combined organic fractions were washed with water and brine, dried over anhydrous MgSO_4_ and evaporated under reduced pressure. The residue was purified by flash column chromatography on silica gel using eluents (petroleum ether/ethyl acetate = 10/1) to afford lansiumamide B (**2**) as yellowish oil, which would solidify after storage in the refrigerator (1.03 g, 95% yield). In order to obtain the analytical sample, recrystalization was carried out with diethyl ether and yellowish plates were secured. Mp = 73–74 °C. (lit. 72–73 °C) [7] ^1^H NMR (400 MHz, CDCl_3_) δ: 7.63 (d, *J* = 15.6 Hz, 1H), 7.47–7.44 (m, 2H), 7.34–7.28 (m, 7H), 7.25–7.20 (m, 1H), 6.93 (d, *J* = 15.6 Hz, 1H), 6.50 (d, *J* = 8.4 Hz, 1H), 6.24 (d, *J* = 8.4 Hz, 1H), 3.09 (s, 3H). ^13^C NMR (100 MHz, CDCl_3_) δ: 166.6, 142.8, 135.3, 134.5, 129.8, 128.9, 128.8 (2×C), 128.7 (4×C), 128.2, 128.1 (2×C), 125.2, 118.4, 34.8. Anal. calcd for C_18_H_17_NO: C, 82.10; H, 6.51; N, 5.32. Found: C, 82.18; H, 6.58; N, 5.23.

### 3.3. Total Synthesis of Alatamide

#### 3.3.1. Synthesis of (*E*)-1-(2-iodovinyl)-4-methoxybenzene (**9**)

4-Methoxy-cinnamic acid (**8**) (1.43 g, 8 mmol) was dissolved in dichloromethane (12 mL). Subsequently, Et_3_N (0.23 mL, 1.6 mmol) was added followed by the addition of *N*-iodosuccinamide (NIS) (2.16 g, 9.6 mmol), and the reaction was allowed to run for 30 min. After the completion of reaction, the reaction mixture was evaporated under reduced pressure at a low temperature. The residue was purified by flash column chromatography on silica gel using eluents (petroleum ether/ethyl acetate = 20/1) to afford (*E*)-1-(2-iodovinyl)-4-methoxybenzene (**9**) as brown solid (1.40 g, 67% yield). Mp = 99–100 °C. (lit. 98–99 °C) [25] ^1^H NMR (400 MHz, CDCl_3_) δ: 7.36 (d, *J* = 14.8 Hz, 1H), 7.23 (dd, *J* = 6.8, 2.0 Hz, 1H), 6.85 (dd, *J* = 6.8, 2.0 Hz, 1H), 6.63 (d, *J* = 14.8 Hz, 1H), 3.81 (s, 3H). ^13^C NMR (100 MHz, CDCl_3_) δ: 159.8, 144.4, 130.7, 127.4 (2×C), 114.1 (2×C), 73.8, 55.4.

#### 3.3.2. Synthesis of Alatamide (**7**)

A Schlenk tube was charged with CuI (48 mg, 0.25 mmol, and 5 mol%), Cs_2_CO_3_ (2.44 g, 7.5 mmol) and benzamide (0.726 g, 6.0 mmol), and evacuated and backfilled with argon. *N*,*N*′-Dimethylethylenediamine (54 μL, 0.50 mmol, and 10 mol%), (*E*)-1-(2-iodovinyl)-4-methoxybenzene (**9**) (1.30 g, 5.0 mmol) and THF (10.0 mL) were added under argon. The Schlenk tube was sealed, immersed in a preheated oil bath; the reaction mixture was stirred at 60 °C until the complete consumption of starting material was observed by TLC (5 h). The reaction vessel was removed from the oil bath and the resulting suspension was allowed to reach room temperature, then, it was filtered through a plug silica gel eluting with ethyl acetate (50 mL). The filtrate was concentrated and the residue was purified by flash chromatography on silica gel using eluents (petroleum ether/ethyl acetate = 5/1) to provide alatamide (**7**) as white solid (1.06 g, 84% yield). Mp = 176–178 °C. (lit. 178–180 °C) [19] ^1^H NMR (400 MHz, CDCl_3_) δ: 7.95 (br, 1H), 7.85–7.82 (m, 2H), 7.61 (dd, *J* = 14.8, 10.8 Hz, 1H), 7.56–7.52 (m, 1H), 7.49–7.44 (m, 2H), 7.30–7.26 (m, 2H), 6.86–6.82 (m, 2H), 6.22 (d, *J* = 14.8 Hz, 1H), 3.80 (s, 3H). ^13^C NMR (100 MHz, CDCl_3_) δ: 164.3, 158.6, 133.5, 132.0, 128.7 (2×C), 128.5, 127.0 (2×C), 126.7 (2×C), 121.3, 114.1 (2×C), 113.3, 55.2. Anal. calcd for C_16_H_15_NO_2_: C, 75.87; H, 5.97; N, 5.53. Found: C, 75.80; H, 6.13; N, 5.38.

## 4. Conclusions

We have accomplished the total synthesis of lansiumamides A, B and alatamide in three steps with a 48% yield from phenylacetylene, four steps with a 46% yield from phenylacetylene and two steps with a 56% yield from 4-methoxycinnamic acid, respectively, by taking advantage of the copper-catalyzed Buchwald coupling. The syntheses are concise, convergent, practical and can be carried out on a one-gram scale. This approach could give either *Z* or *E*-configured enamide moiety in natural products with absolute stereocontrol. Further exploration of the strategy for the generation of novel analogues for biological evaluation is ongoing in our laboratory.

## Supplementary Materials

The following are available online. Copies of ^1^H, ^13^C, spectra of new compounds.
