# Supplementary material for: Concise and Gram-Scale Total Synthesis of Lansiumamides A and B and Alatamide
Source: Molecules. 2019 Oct 19;24(20):3764. doi: 10.3390/molecules24203764 (PMC6832413; doi:10.3390/molecules24203764)
Supplement: Supplementary file 1 [file molecules-24-03764-s001.pdf]

Supporting Information

Concise and Gram-Scale Total Synthesis of Lansiumamides A and B and Alatamide

Ran Lin, Xi Lin, Qian Su, Binbin Guo, Yanqin Huang, Ming-An Ouyang, Liyan Song * and Huiyou Xu *

Key Laboratory of Biopesticide and Chemical Biology, Ministry of Education, Fujian Agriculture and Forestry University, Fuzhou, China

songliyan@fafu.edu.cn, huiyouxu@126.com

Table of Content

^{1}H and ^{13}C NMR Spectra of New Compounds **S-2**

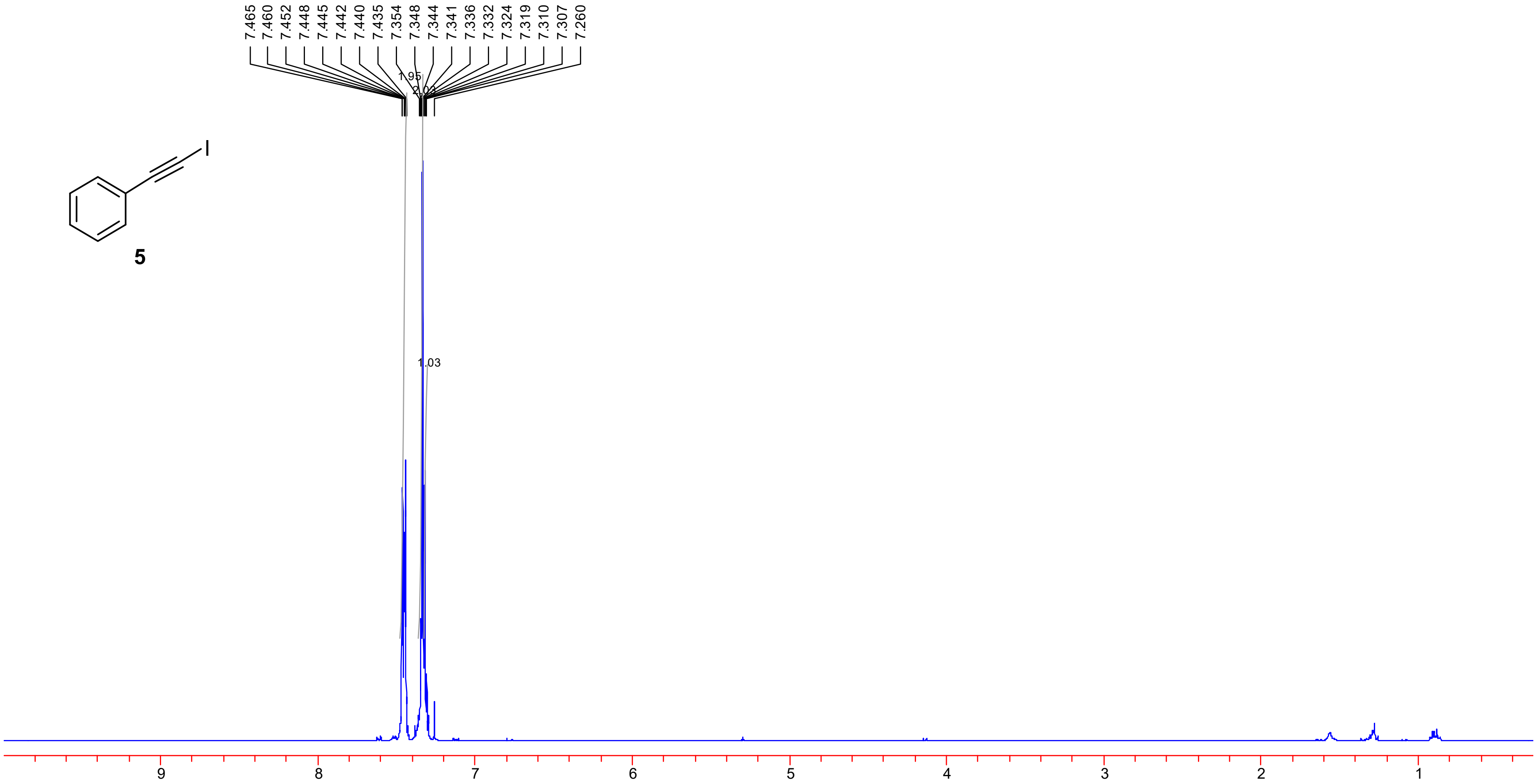
I
5
7.465
7.460
7.452
7.448
7.445
7.442
7.440
7.435
7.354
7.348
7.344
7.341
7.336
7.332
7.324
7.319
7.310
7.307
7.260
1.95
2.01
1.03
9
8
7
6
5
4
3
2
1

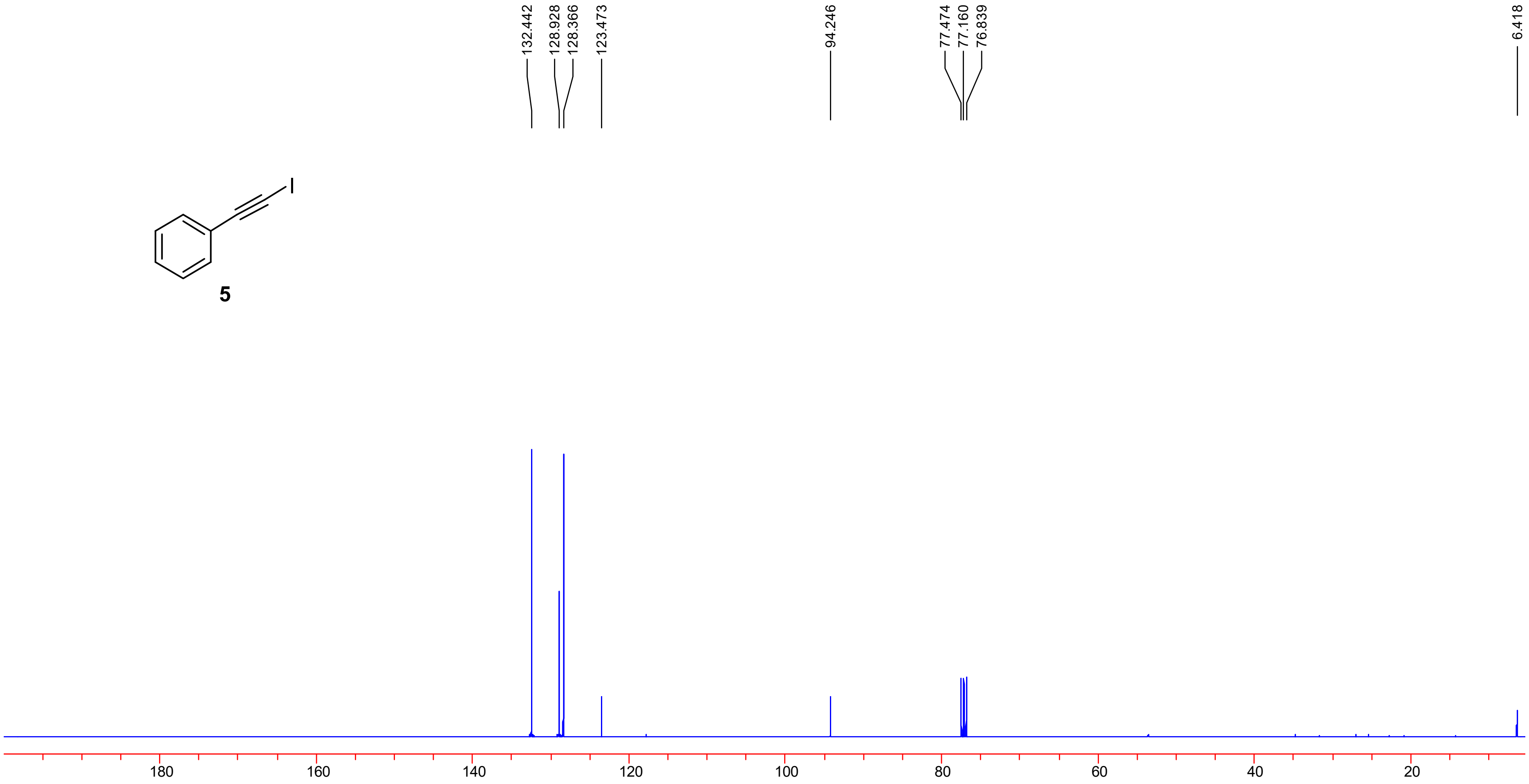
I
132.442
128.928
128.366
123.473
94.246
77.474
77.160
76.839
6.418
180
160
140
120
100
80
60
40
20

5

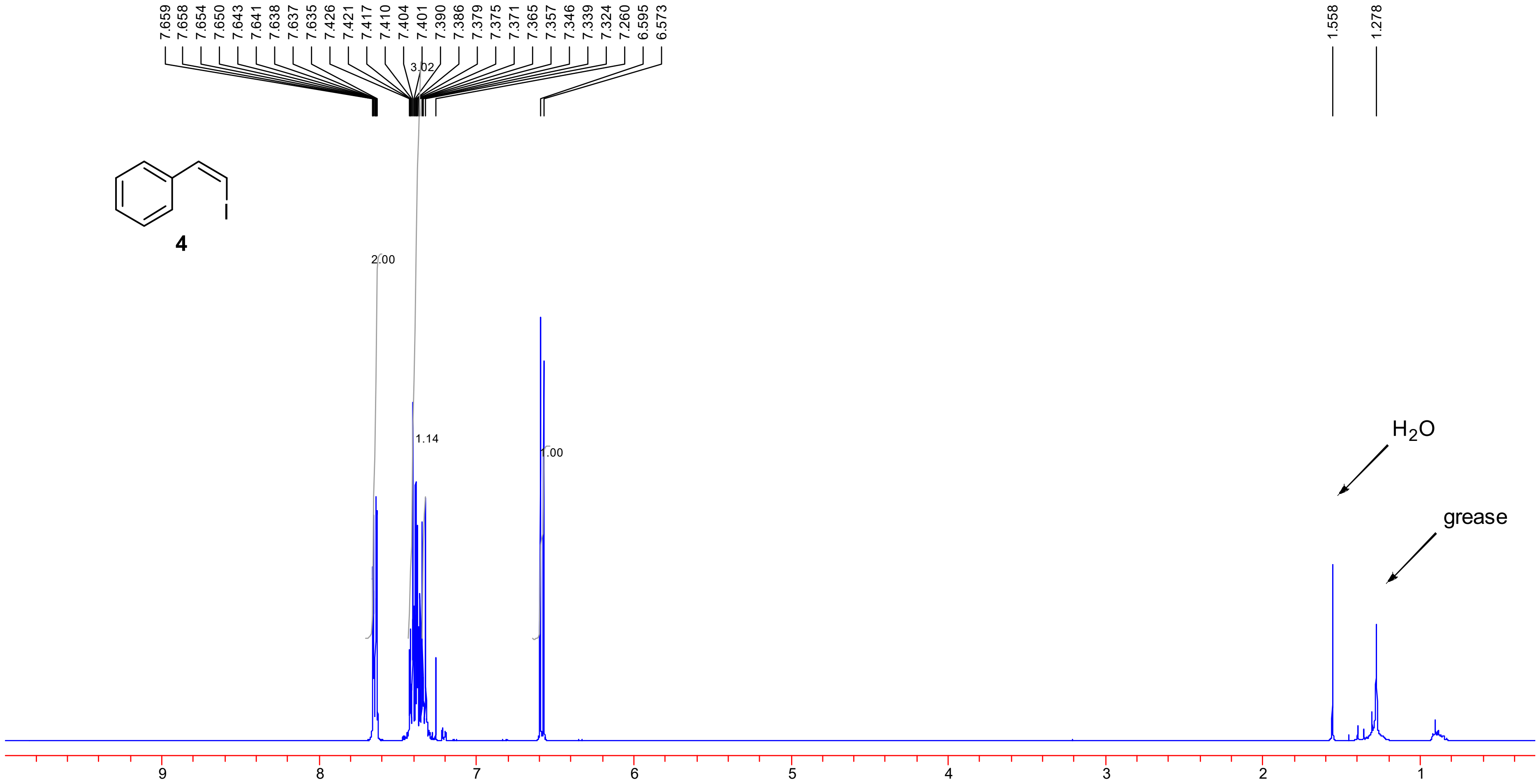
7.659
7.658
7.654
7.650
7.643
7.641
7.638
7.637
7.635
7.426
7.421
7.417
7.410
7.404
7.401
7.390
7.386
7.379
7.375
7.371
7.365
7.357
7.346
7.339
7.324
7.260
6.595
6.573
1.558
1.278
I
4
2.00
3.02
1.14
1.00
H2O
grease
9
8
7
6
5
4
3
2
1

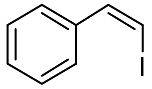

4

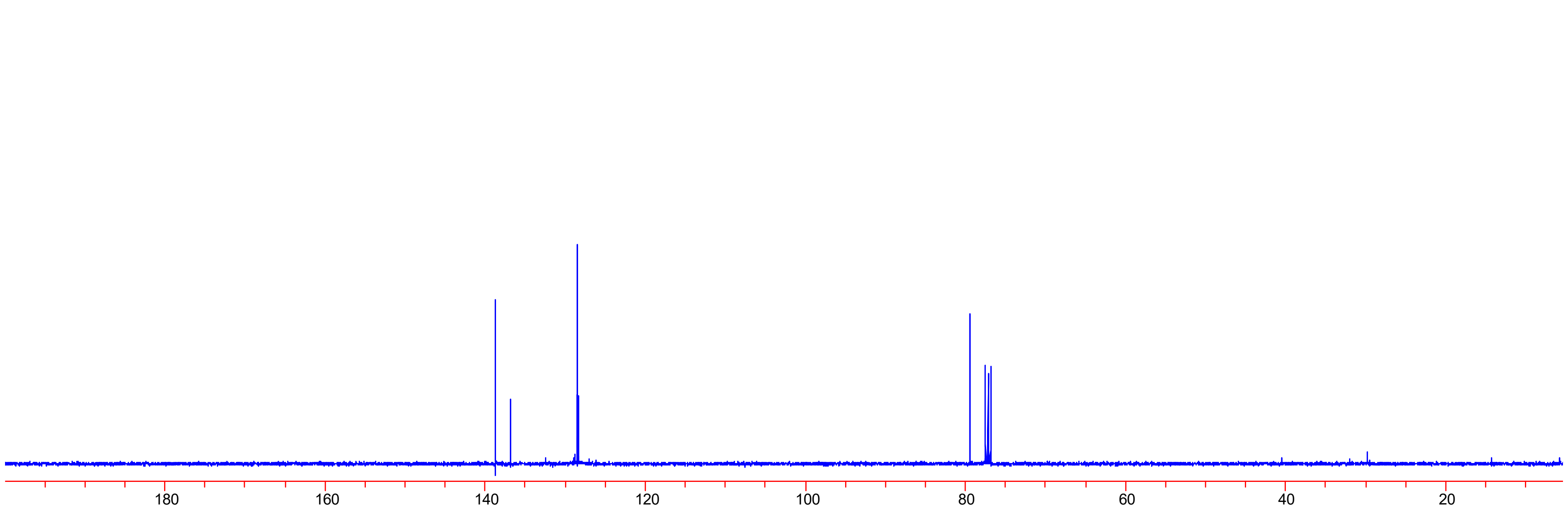
138.693
136.812
128.521
128.492
128.317
79.479
77.481
77.160
76.846
180
160
140
120
100
80
60
40
20

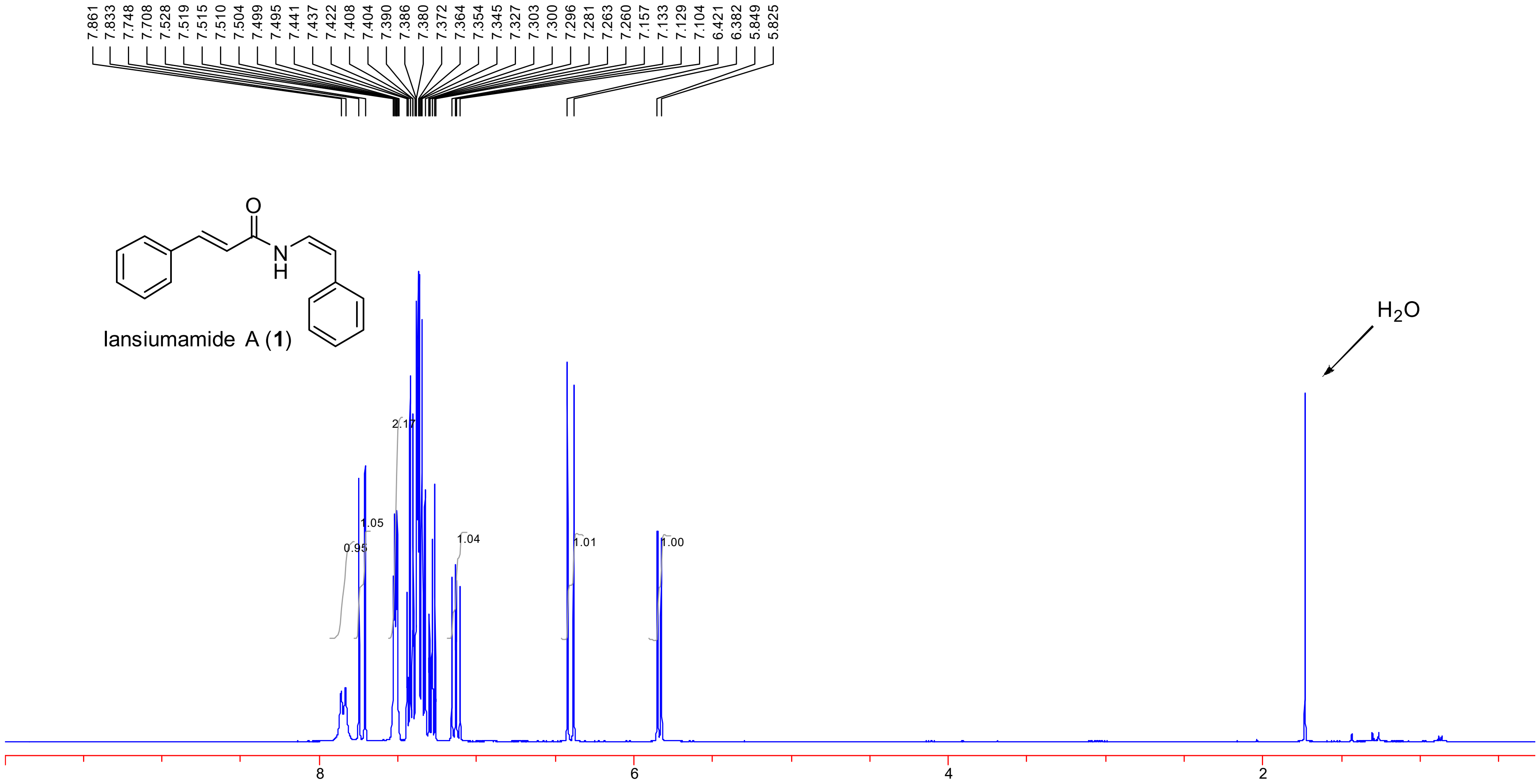
7.861
7.833
7.748
7.708
7.528
7.519
7.515
7.510
7.504
7.499
7.495
7.441
7.437
7.422
7.408
7.404
7.390
7.386
7.380
7.372
7.364
7.354
7.345
7.327
7.303
7.300
7.296
7.281
7.263
7.260
7.157
7.133
7.129
7.104
6.421
6.382
5.849
5.825
O
N
H
lansiumamide A (**1**)
H_2O
0.95
1.05
2.17
1.04
1.01
1.00
8
6
4
2

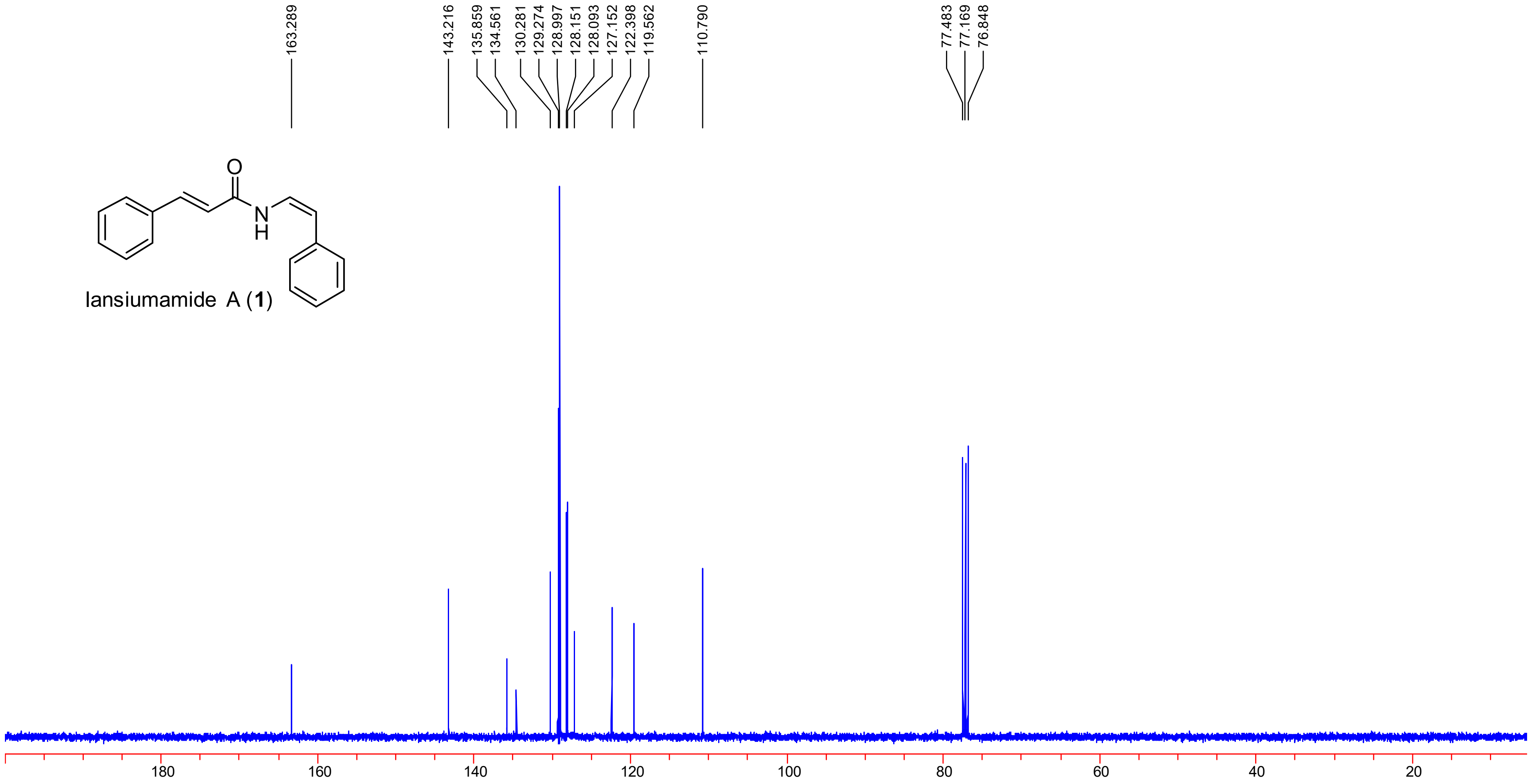

163.289
143.216
135.859
134.561
130.281
129.274
128.997
128.151
128.093
127.152
122.398
119.562
110.790
77.483
77.169
76.848
O
N
H
lansiumamide A (**1**)
180
160
140
120
100
80
60
40
20

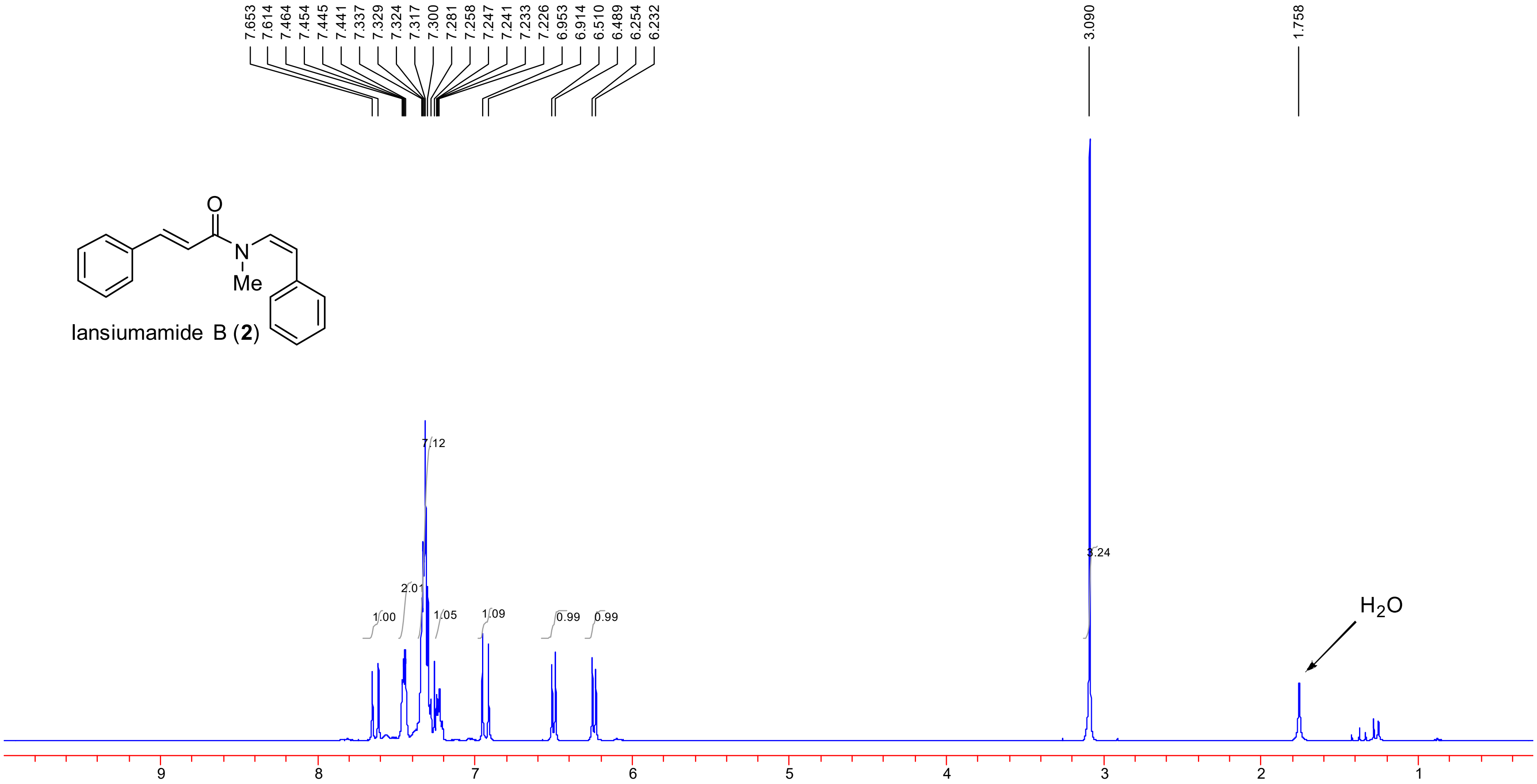
7.653
7.614
7.464
7.454
7.445
7.441
7.337
7.329
7.324
7.317
7.300
7.281
7.258
7.247
7.241
7.233
7.226
6.953
6.914
6.510
6.489
6.254
6.232
3.090
1.758
O
N
Me
lansiumamide B (2)
1.00
2.01
7.12
1.05
1.09
0.99
0.99
3.24
H_2O
9
8
7
6
5
4
3
2
1

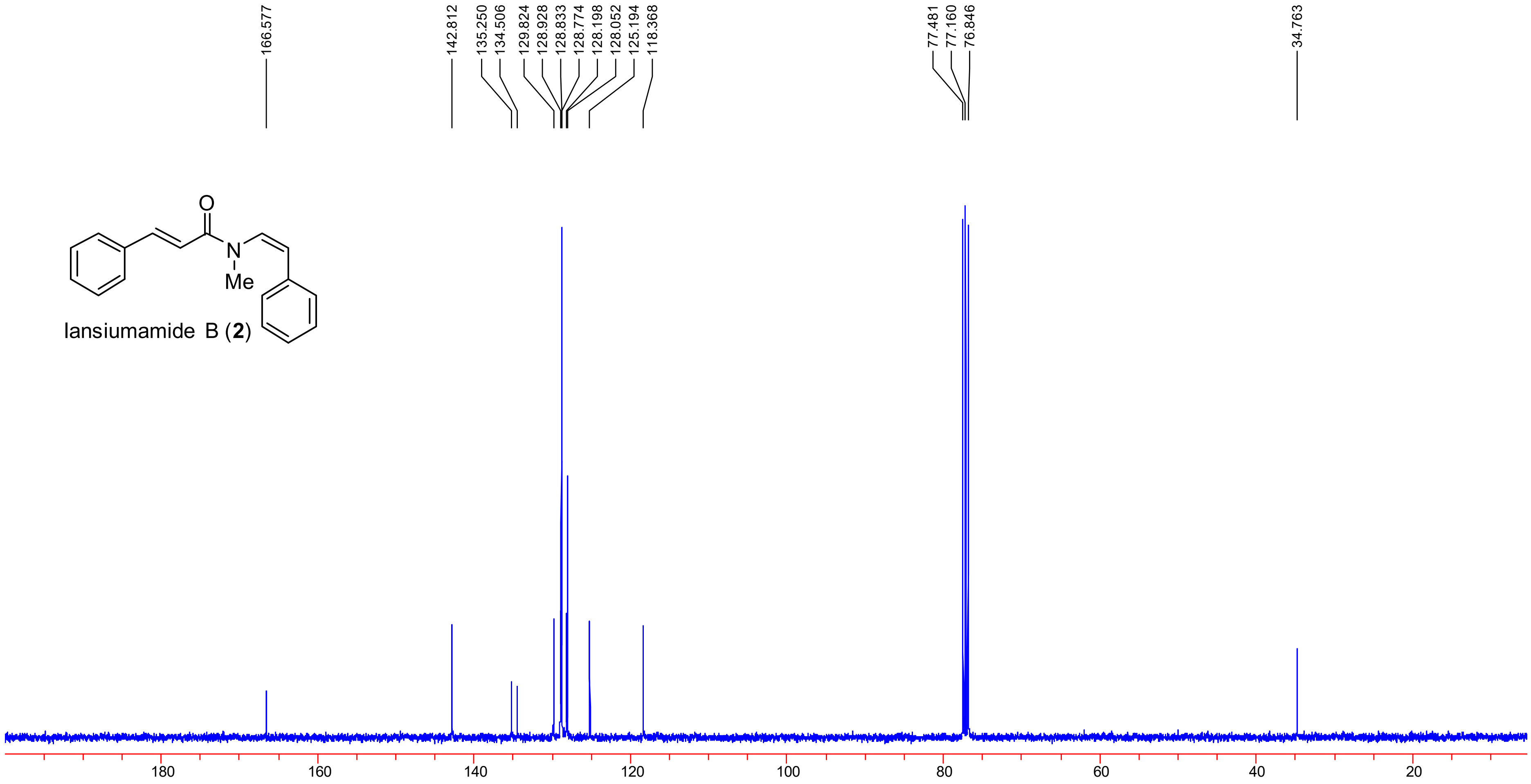
166.577
142.812
135.250
134.506
129.824
128.928
128.833
128.774
128.198
128.052
125.194
118.368
77.481
77.160
76.846
34.763
O
N
Me
lansiumamide B (2)
180
160
140
120
100
80
60
40
20

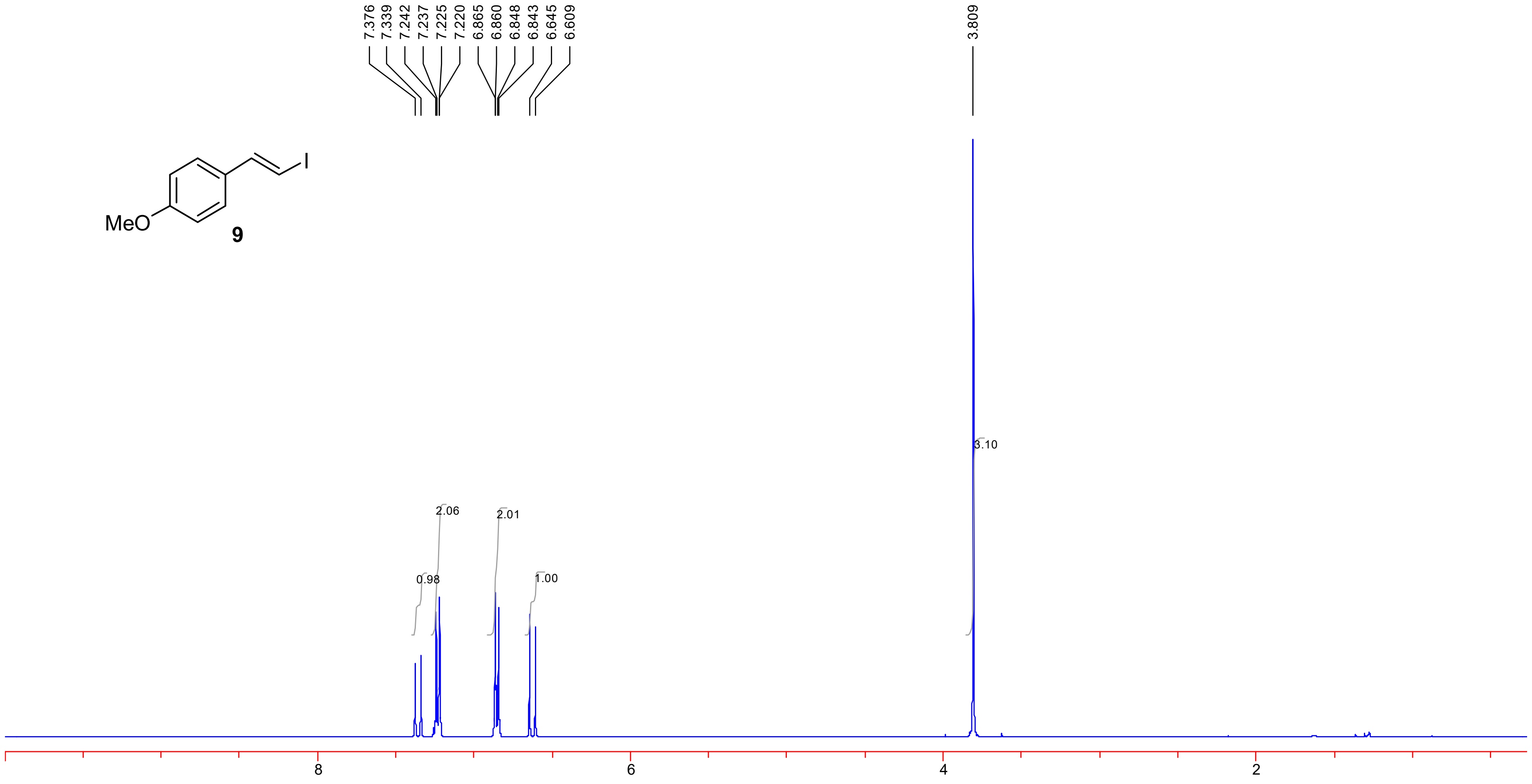
7.376
7.339
7.242
7.237
7.225
7.220
6.865
6.860
6.848
6.843
6.645
6.609
3.809
MeO
I
9
0.98
2.06
2.01
1.00
3.10
8
6
4
2

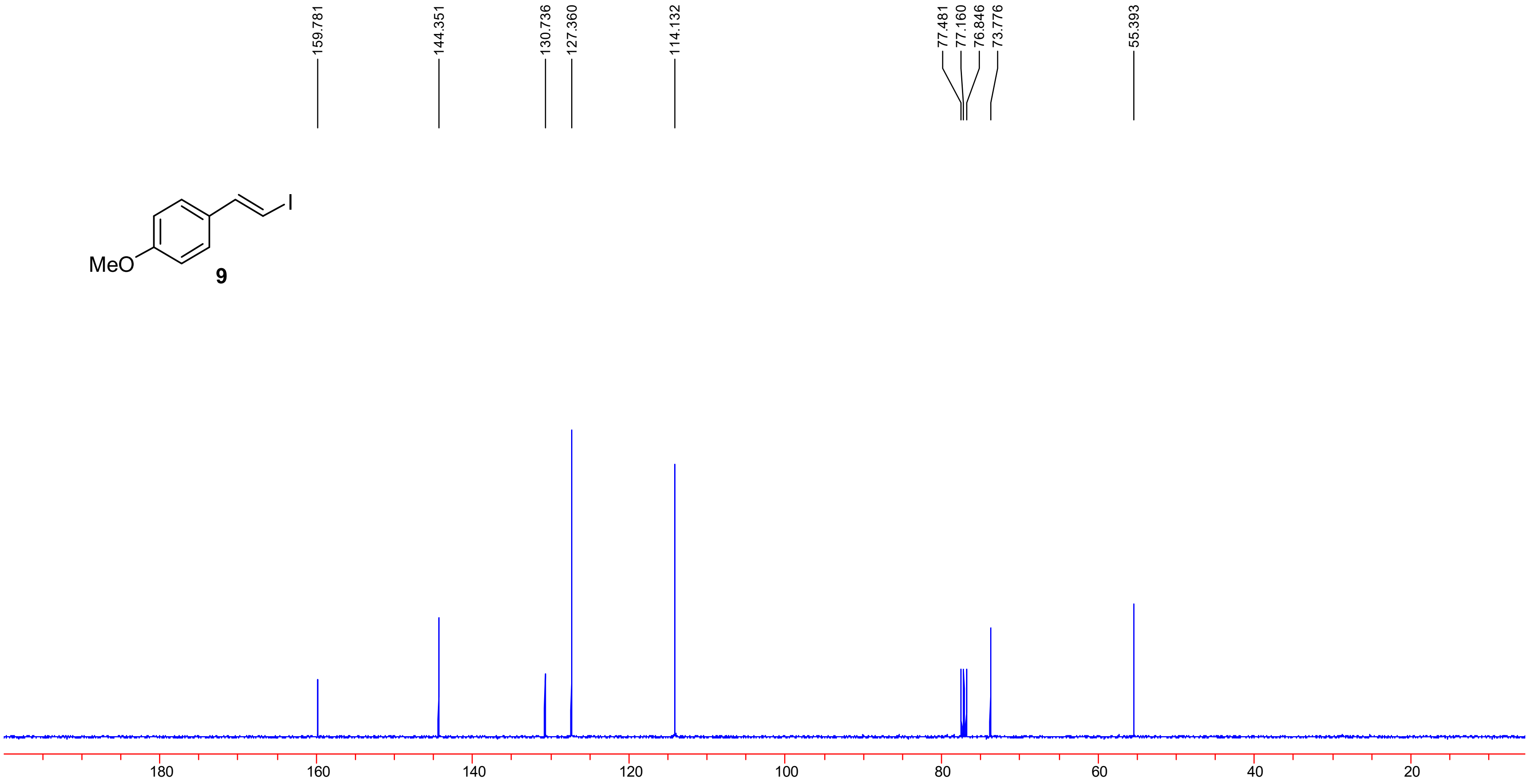
159.781
144.351
130.736
127.360
114.132
77.481
77.160
76.846
73.776
55.393
MeO
I
9
180
160
140
120
100
80
60
40
20

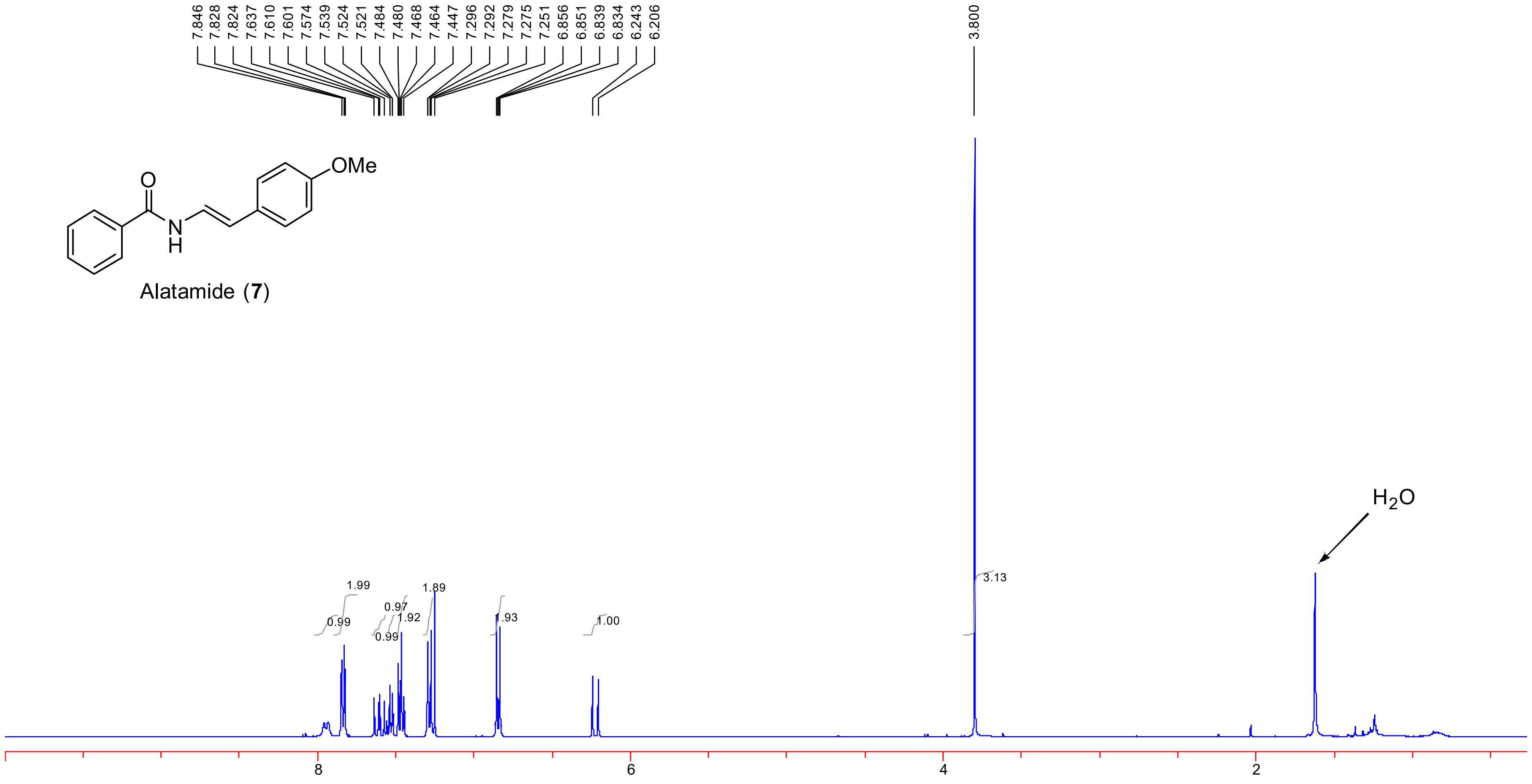

7.846
7.828
7.824
7.637
7.610
7.601
7.574
7.539
7.524
7.521
7.484
7.480
7.468
7.464
7.447
7.296
7.292
7.279
7.275
7.251
6.856
6.851
6.839
6.834
6.243
6.206
3.800
OMe
O
N
H
Alatamide (7)
0.99
1.99
0.99
0.97
1.92
1.89
1.93
1.00
3.13
H2O
8
6
4
2

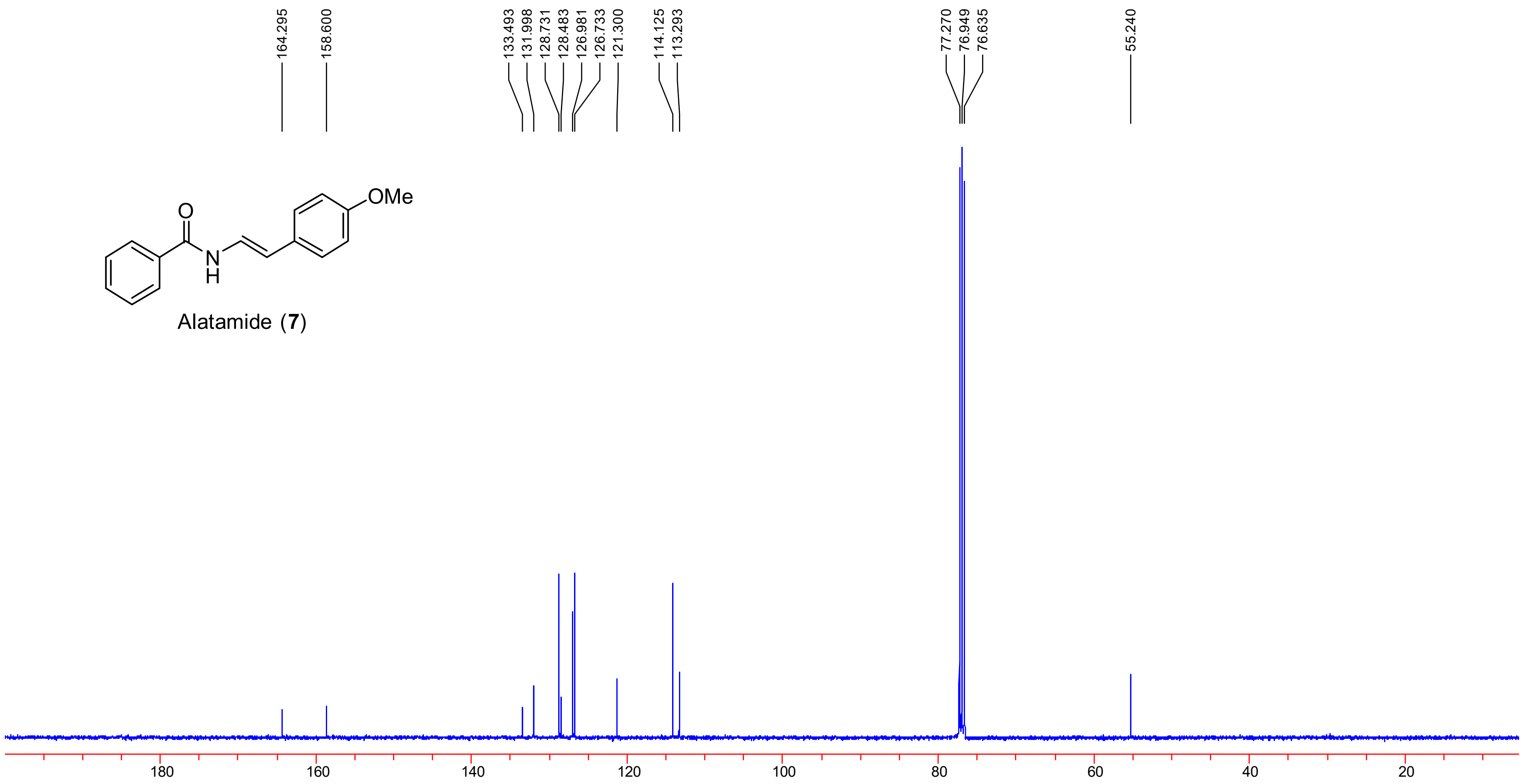
164.295
158.600
133.493
131.998
128.731
128.483
126.981
126.733
121.300
114.125
113.293
77.270
76.949
76.635
55.240
OMe
O
N
H
Alatamide (7)
180
160
140
120
100
80
60
40
20
